# Mortality risk following ischaemic and non‐ischaemic heart failure in people with type 2 diabetes: Observational study in England, 2000–2021

**DOI:** 10.1111/dom.16413

**Published:** 2025-05-02

**Authors:** Kajal Panchal, Claire A. Lawson, Sharmin Shabnam, Kamlesh Khunti, Francesco Zaccardi

**Affiliations:** ^1^ Leicester Real World Evidence Unit, Leicester Diabetes Centre University of Leicester Leicester UK; ^2^ Department of Cardiovascular Sciences University of Leicester Leicester UK

**Keywords:** cohort study, diabetes complications, heart failure, population study, real‐world evidence, type 2 diabetes

## Abstract

**Aims:**

To investigate the association between type 2 diabetes and all‐cause mortality in people with ischaemic and non‐ischaemic heart failure (HF).

**Methods:**

Using the Clinical Practice Research Datalink primary care data, linked to hospital and mortality records, we identified newly diagnosed adults with type 2 diabetes between 2000 and 2021 and matched to up to four people without diabetes by sex, year of birth and general practice. We defined incident HF events as ischaemic if they followed an ischaemic heart disease event; otherwise, as non‐ischaemic. We calculated sex‐specific incidence rates and hazard ratios (HRs, adjusted for sociodemographic and clinical confounders) for all‐cause mortality following HF diagnosis, comparing people with type 2 diabetes to those without diabetes.

**Results:**

In 73 344 people with HF (18 296 [24.9%] with ischaemic HF), 9584 and 31 800 deaths occurred in those with ischaemic and non‐ischaemic HF, respectively. Age‐standardised mortality rates following ischaemic HF were higher in people with type 2 diabetes (19.2 [95% CI: 18.1–20.3] and 20.4 [19.5–21.4] per 100 person‐years in women and men, respectively) compared to those without diabetes (15.1 [14.4–15.8] and 16.5 [15.9–17.1], respectively). Corresponding rates in those with non‐ischaemic HF were: 19.5 (19.0–20.1), 22.0 (21.4–22.6), 16.6 (16.2–17.0) and 19.4 (18.9–19.8). The HR for mortality was similar among HF types and between men and women: for ischaemic HF, 1.26 (1.17–1.36) in women and 1.23 (1.15–1.31) in men; for non‐ischaemic HF, 1.24 (1.19–1.29) and 1.20 (1.15–1.25), respectively.

**Conclusion:**

Our findings indicate broadly similar mortality rates in people with ischaemic and non‐ischaemic HF, with higher rates in people with type 2 diabetes compared to those without diabetes in both women and men.

## 1 INTRODUCTION

Diabetes mellitus and heart failure (HF) are complex chronic diseases and they commonly co‐exist^1^; recent global data suggest an estimated ~540 million^2^ and ~64 million people^3^ are impacted by these conditions, respectively. Notably, 20%–40% of people with HF have type 2 diabetes,^4^ and in England alone, diabetes leads to approximately 2300 new cases of HF per week.^5^

Observational studies have recognised diabetes as a major risk factor for mortality among people with HF,^6^, ^7^ while other studies have shown similar mortality risk in people with HF, regardless of diabetes status.^8^, ^9^ Nonetheless, recent advancements in cardiovascular disease prevention strategies^10^, ^11^ have resulted in parallel decreasing trends in HF^12^, ^13^ and ischaemic heart diseases (IHD)^14^ over time in the United Kingdom. However, it remains unclear whether, and to what extent, these changes might have impacted the mortality risk among people with diabetes and HF in relation to its aetiology, that is ischaemic or non‐ischaemic. This is necessary to aid the development of more tailored prevention and management strategies aimed at reducing the mortality risk in people with HF.

Most epidemiological studies investigating the mortality risk in people with HF by aetiology and diabetes status report higher risk in people with ischaemic compared to non‐ischaemic HF, and higher in people with versus those without diabetes,^15^, ^16^, ^17^, ^18^, ^19^ with one contradictory finding.^20^ However, many studies in this area relined on clinical trials^15^, ^18^ or hospital data,^19^, ^20^ with limited generalisability to the general population. Moreover, few of these studies have objectively defined diabetes as ‘type 2 diabetes’,^8^, ^17^ a distinction necessary to provide greater detail. Additionally, the majority of studies investigating mortality risk among people with HF of ischaemic and non‐ischaemic aetiology by diabetes status are from earlier time periods,^15^, ^16^, ^17^, ^18^, ^19^ with the latest data on this topic being reported up to 2011.^17^, ^19^ This highlights a clear need for more up‐to‐date research, especially in view of recent advancements in HF therapies, such as sodium–glucose cotransporter 2 inhibitors and glucagon‐like peptide‐1 receptor agonists.^10^, ^11^ There is also a scarcity of UK studies investigating mortality risk among people with HF by aetiology and diabetes status.^19^

Therefore, the current study aimed to investigate the mortality risk following ischaemic and non‐ischaemic HF in people with type 2 diabetes and without diabetes between 2000 and 2021 using primary care and hospital data from England.

## 2 METHODS

### 2.1 Data sources

This observational retrospective cohort study used data from the Clinical Practice Research Datalink (CPRD) databases (GOLD and Aurum) linked to the Hospital Episode Statistics Admitted Patient Care (HES APC) data and the Office for National Statistics (ONS) death registration data in England. The CPRD is an electronic health record database, capturing information on primary care electronic patient records including demographic, clinical, laboratory and prescription records (https://www.cprd.com/) and is representative of the UK population in relation to age, sex and ethnicity.^21^, ^22^ This study was conducted in line with the RECORD guidelines (checklist reported in the Data S1) and was approved by an Independent Scientific Advisory Committee (protocol: 21_000355) prior to conducting the analysis.

### 2.2 Study population

We firstly identified all adults (≥18 years) with newly diagnosed type 2 diabetes in CPRD GOLD and Aurum between 1st January 2000 and 29th March 2021, who were matched with up to 4 people without diabetes based on sex, birth year and general practice. All individuals were required to have been registered for at least 12 months prior to their first recorded date of diagnosis of type 2 diabetes (or corresponding matched index date for those without diabetes), to meet acceptable research standards and to have available linkage with HES APC and ONS.

We then identified individuals with a first recorded diagnosis code for incident HF after their type 2 diabetes diagnosis date (or matched date for those without diabetes), and we included them in our study. Individuals with a prior history of cardiovascular disease (IHD, peripheral vascular disease, stroke) or HF on or before the type 2 diabetes diagnosis date (or corresponding matched date for those without diabetes) were excluded. Detailed flowcharts of the study population definition are reported in Figure S1 (GOLD) and Figure S2 (Aurum).

### 2.3 Main exposure and covariates

In people with ischaemic and non‐ischaemic HF, the primary exposure of interest was type 2 diabetes. Ischaemic and non‐ischaemic HF were defined based on the first diagnosis code in CPRD GOLD, CPRD Aurum or HES APC after the diagnosis date of type 2 diabetes (or the corresponding date for the matched individual(s) without diabetes). Ischaemic HF events were defined as HF episodes with a prior diagnosis of IHD (coronary heart disease, angina, myocardial infarction, coronary artery bypass graft surgery, percutaneous coronary intervention) from the diagnosis date of type 2 diabetes (corresponding date for those without diabetes) until 29th March 2021 (last ONS linkage date), while non‐ischaemic HF events were defined as HF episodes without a prior diagnosis of IHD.

Covariates included age (years), sex (female, male), ethnicity (White, Black, South Asian, Mixed/Other, Unknown – defined using HES) and deprivation (measured in quintiles of the Index of Multiple Deprivation, a proxy for individual‐level socioeconomic status^23^; IMD 1st, least deprived; 5th, most deprived). We extracted information on the following cardiovascular risk factors: smoking status (ever‐smoker, never‐smoker), alcohol consumption (current, ex, never), comorbidities (anaemia, asthma, atrial fibrillation, cancer, chronic kidney disease, chronic liver disease, chronic obstructive pulmonary disease, dementia, depression, hypertension, osteoarthritis, rheumatoid arthritis and thyroid disorders defined in CPRD GOLD, CPRD Aurum or HES), medication prescriptions (antiplatelet, antihypertensive and lipid‐lowering medications, digoxin), body mass index (BMI), systolic blood pressure and total cholesterol. Information on all variables was extracted from the latest available record prior to or the same date as the first diagnosis date of incident HF.

### 2.4 Outcome

The outcome of interest was all‐cause mortality, defined using the ONS database. Follow‐up was until death or the last ONS linkage date (29th March 2021).

### 2.5 Statistical analysis

We summarised the baseline characteristics at HF diagnosis stratified by diabetes status, sex and HF aetiology (ischaemic and non‐ischaemic) as number (proportion) for categorical data and mean (SD) for continuous data. The baseline characteristics comparing people with type 2 diabetes vs. without diabetes, stratified by sex and HF aetiology, were compared using the independent *t*‐test (for continuous data – normally distributed), Mann–Whitney (for continuous data – nonnormally distributed or ordinal categorical data) or chi‐squared test (for nominal categorical data) as appropriate. We used Poisson regression models to calculate sex‐specific crude and age‐standardised mortality rates (at the mean age of the total population, 78 years old) following the ischaemic and non‐ischaemic HF episode by diabetes status. Poisson regression models were also used to estimate sex‐stratified crude and age‐adjusted incidence rate ratios (IRRs) of mortality comparing people with type 2 diabetes vs. those without diabetes.

We used complete‐case Royston–Parmar survival models to estimate sex‐specific hazard ratios (HRs), comparing those with type 2 diabetes to those without diabetes, with the following adjustments: Model 1, unadjusted; Model 2, adjusted for age; Model 3, Model 2 + IMD, ethnicity, smoking, alcohol intake; Model 4, Model 3 + comorbidities, BMI, systolic blood pressure, total cholesterol level; Model 5, Model 4 + medications. A likelihood ratio test was used to compare the maximally adjusted Royston–Parmar survival model (Model 5) with and without an interaction term between diabetes status and sex; this approach assessed whether associations between diabetes and all‐cause mortality varied by sex; a *p*‐value of <0.05 was considered statistically significant. Data preparation and analyses were performed in Stata/BE 17.0, Stata/BE 18.0 and Python 3.8.

### 2.6 Sensitivity analysis

We repeated all analyses excluding those with prevalent HF, peripheral vascular disease or stroke and including those with prevalent IHD prior to or at the diagnosis date of type 2 diabetes (or the corresponding date in those without diabetes). This allowed us to define ischaemic HF as an incident HF episode with prior diagnosis of IHD at any time in the study period – including before the diagnosis date of type 2 diabetes. Furthermore, we also repeated all analyses stratified by the date of incident HF diagnosis (2000–2010; 2011–2021) to assess whether our results differed according to calendar period of diagnosis.

## 3 RESULTS

### 3.1 Baseline characteristics

The final cohort included 73 344 people with HF, of which 18 296 (24.9%) had ischaemic and 55 048 had non‐ischaemic HF (Table 1, Tables S1–S3). Among those with ischaemic HF, 2806 women (15.3%) and 4447 men (24.3%) had type 2 diabetes; corresponding figures for non‐ischaemic HF were 10 094 (18.3%) and 9964 (18.1%) (Table 1). Among people with ischaemic HF, compared to those without diabetes, both women and men with type 2 diabetes were less likely to be of White ethnicity and more likely to be in the most deprived quintile; this pattern was observed also for non‐ischaemic HF. Regardless of incident HF aetiology, both women and men with type 2 diabetes had higher mean BMI (~3.02 to 4.27 kg/m^2^ higher), lower mean total cholesterol (~0.30 to 0.44 mmol/L lower), higher prevalence of comorbidities (i.e. anaemia, atrial fibrillation, depression, hypertension and valvular heart disease) and higher medication prescriptions (i.e. antihypertensive, antiplatelet, glucose‐lowering and lipid‐lowering drugs) compared to those without diabetes (Table 1).

**TABLE 1:** Sex‐stratified baseline characteristics at heart failure diagnosis.

	Ischaemic heart failure (*n* = 18 296)						Non‐ischaemic heart failure (*n* = 55 048)					
	Women (*n* = 7117)			Men (*n* = 11 179)			Women (*n* = 28 015)			Men (*n* = 27 033)		
	Type 2 diabetes (*n* = 2806)	Without diabetes (*n* = 4311)	*p*‐value	Type 2 diabetes (*n* = 4447)	Without diabetes (*n* = 6732)	*p*‐value	Type 2 diabetes (*n* = 10 094)	Without diabetes (*n* = 17 921)	*p*‐value	Type 2 diabetes (*n* = 9964)	Without diabetes (*n* = 17 069)	*p*‐value
Age (years)	77.26 ± 10.91	80.90 ± 8.78	0.000	73.44 ± 10.96	76.60 ± 9.72	0.000	78.33 ± 11.19	81.15 ± 9.08	0.000	74.83 ± 11.65	77.38 ± 9.97	0.000
Year of incident HF diagnosis (median, IQR)	2016 (2012–2019)	2016 (2012–2018)	0.964	2016 (2012–2018)	2016 (2012–2018)	0.495	2015 (2011–2018)	2016 (2012–2018)	0.000	2016 (2011–2018)	2016 (2012–2018)	0.140
Ethnicity												
White	2414 (86.03)	4046 (93.85)	0.000	3869 (87.00)	6284 (93.35)	0.000	8858 (87.77)	16 537 (92.28)	0.000	8882 (89.14)	15 788 (92.50)	0.000
South Asian	197 (7.02)	94 (2.18)		279 (6.27)	154 (2.29)		405 (4.01)	336 (1.87)		269 (2.70)	222 (1.30)	
Black	76 (2.71)	58 (1.35)		102 (2.29)	87 (1.29)		378 (3.74)	349 (1.95)		341 (3.42)	339 (1.99)	
Mixed/Other	90 (3.21)	58 (1.35)		136 (3.06)	106 (1.57)		221 (2.19)	269 (1.50)		225 (2.26)	263 (1.54)	
Unknown	29 (1.03)	55 (1.28)		61 (1.37)	101 (1.50)		231 (2.29)	430 (2.40)		247 (2.48)	457 (2.68)	
IMD quintile												
1 (least deprived)	447 (15.93)	775 (17.98)	0.000	753 (16.93)	1370 (20.35)	0.000	1580 (15.65)	3373 (18.82)	0.000	1775 (17.81)	3546 (20.77)	0.000
2	474 (16.89)	840 (19.49)		842 (18.93)	1458 (21.66)		1830 (18.13)	3652 (20.38)		1942 (19.49)	3700 (21.68)	
3	524 (18.67)	828 (19.21)		862 (19.38)	1289 (19.15)		2015 (19.96)	3600 (20.09)		1978 (19.85)	3370 (19.74)	
4	610 (21.74)	934 (21.67)		951 (21.39)	1296 (19.25)		2217 (21.96)	3659 (20.42)		2055 (20.62)	3270 (19.16)	
5 (most deprived)	751 (26.76)	934 (21.67)		1039 (23.36)	1319 (19.59)		2452 (24.29)	3637 (20.29)		2214 (22.22)	3183 (18.65)	
Smoking status												
Ever‐smoker	1300 (46.33)	1970 (45.70)	0.601	2895 (65.10)	4187 (62.20)	0.002	4528 (44.86)	7499 (41.84)	0.000	6391 (64.14)	10 206 (59.79)	0.000
Nonsmoker	1506 (53.67)	2341 (54.30)		1552 (34.90)	2545 (37.80)		5566 (55.14)	10 422 (58.16)		3573 (35.86)	6863 (40.21)	
Alcohol intake												
Current	2212 (82.51)	3298 (83.45)	0.490	3761 (89.33)	5525 (90.78)	0.011	7953 (83.07)	13 515 (83.40)	0.000	8529 (90.69)	13 899 (91.58)	0.036
Ex‐drinker	37 (1.38)	45 (1.14)		83 (1.97)	81 (1.33)		144 (1.50)	155 (0.96)		145 (1.54)	193 (1.27)	
Never	432 (16.11)	609 (15.41)		366 (8.69)	480 (7.89)		1477 (15.43)	2536 (15.65)		731 (7.77)	1085 (7.15)	
BMI (kg/m^2^)	30.93 ± 7.27	27.03 ± 6.15	0.000	30.36 ± 5.98	27.34 ± 4.80	0.000	31.99 ± 8.37	27.72 ± 6.73	0.000	31.46 ± 7.10	27.70 ± 5.40	0.000
HbA1c (mmol/mol)	57.03 ± 17.84	39.65 ± 4.34	0.000	56.80 ± 16.73	39.74 ± 5.49	0.000	53.97 ± 15.85	39.65 ± 4.88	0.000	54.65 ± 16.32	39.32 ± 5.25	0.000
Systolic BP (mmHg)	135.51 ± 20.46	134.62 ± 20.32	0.070	131.99 ± 19.22	131.02 ± 19.47	0.010	135.75 ± 19.72	135.64 ± 19.81	0.641	133.77 ± 19.11	133.93 ± 19.15	0.504
Total cholesterol (mmol/l)	4.08 ± 1.35	4.42 ± 1.45	0.000	3.76 ± 1.17	4.06 ± 1.27	0.000	4.05 ± 1.24	4.49 ± 1.42	0.000	3.77 ± 1.12	4.18 ± 1.22	0.000
Comorbidities												
Anaemia	1162 (41.41)	1378 (31.96)	0.000	1408 (31.66)	1656 (24.60)	0.000	3624 (35.90)	5239 (29.23)	0.000	2670 (26.80)	3704 (21.70)	0.000
Asthma	839 (29.90)	1031 (23.92)	0.000	890 (20.01)	1248 (18.54)	0.052	2651 (26.26)	3987 (22.25)	0.000	1963 (19.70)	3274 (19.18)	0.297
Atrial fibrillation	1252 (44.62)	2115 (49.06)	0.000	1882 (42.32)	3231 (47.99)	0.000	4822 (47.77)	9160 (51.11)	0.000	4930 (49.48)	9390 (55.01)	0.000
Cancer	659 (23.49)	1123 (26.05)	0.015	1036 (23.30)	1796 (26.68)	0.000	2657 (26.32)	4909 (27.39)	0.053	2646 (26.56)	5103 (29.90)	0.000
Chronic kidney disease	947 (33.75)	1384 (32.10)	0.148	1158 (26.04)	1650 (24.51)	0.068	3305 (32.74)	5301 (29.58)	0.000	2522 (25.31)	3769 (22.08)	0.000
Chronic liver disease	214 (7.63)	178 (4.13)	0.000	303 (6.81)	302 (4.49)	0.000	696 (6.90)	740 (4.13)	0.000	767 (7.70)	905 (5.30)	0.000
COPD	630 (22.45)	979 (22.71)	0.800	995 (22.37)	1533 (22.77)	0.623	2208 (21.87)	3660 (20.42)	0.004	2392 (24.01)	4228 (24.77)	0.159
Dementia	211 (7.52)	399 (9.26)	0.011	219 (4.92)	367 (5.45)	0.221	864 (8.56)	1766 (9.85)	0.000	561 (5.63)	1100 (6.44)	0.007
Depression	899 (32.04)	1199 (27.81)	0.000	854 (19.20)	1107 (16.44)	0.000	2806 (27.80)	4426 (24.70)	0.000	1724 (17.30)	2651 (15.53)	0.000
Hypertension	2624 (93.51)	3552 (82.39)	0.000	3913 (87.99)	5028 (74.69)	0.000	8817 (87.35)	13 415 (74.86)	0.000	8268 (82.98)	11 320 (66.32)	0.000
Osteoarthritis	1353 (48.22)	2058 (47.74)	0.692	1449 (32.58)	2216 (32.92)	0.713	4677 (46.33)	8111 (45.26)	0.083	3252 (32.64)	5303 (31.07)	0.007
Rheumatoid arthritis	197 (7.02)	332 (7.70)	0.285	160 (3.60)	258 (3.83)	0.522	662 (6.56)	1219 (6.80)	0.434	318 (3.19)	611 (3.58)	0.091
Thyroid disorders	692 (24.66)	990 (22.96)	0.100	428 (9.62)	548 (8.14)	0.007	2333 (23.11)	3753 (20.94)	0.000	804 (8.07)	1247 (7.31)	0.022
Valvular heart disease	1136 (40.48)	1863 (43.22)	0.023	1566 (35.21)	2614 (38.83)	0.000	2903 (28.76)	5495 (30.66)	0.001	2506 (25.15)	4784 (28.03)	0.000
Medication prescription												
Antihypertensive drugs	2735 (97.47)	3931 (91.19)	0.000	4240 (95.35)	5877 (87.30)	0.000	9728 (96.37)	15 737 (87.81)	0.000	9412 (94.46)	14 175 (83.05)	0.000
Antiplatelet drugs	2258 (80.47)	3019 (70.03)	0.000	3615 (81.29)	4758 (70.68)	0.000	5704 (56.51)	7487 (41.78)	0.000	5857 (58.78)	7091 (41.54)	0.000
Digoxin	368 (13.11)	520 (12.06)	0.189	401 (9.02)	605 (8.99)	0.956	1623 (16.08)	2308 (12.88)	0.000	1407 (14.12)	2135 (12.51)	0.000
Glucose‐lowering drugs	2160 (76.98)	–	0.000	3554 (79.92)	–	0.000	6882 (68.18)	–	0.000	7133 (71.59)	–	0.000
Lipid‐lowering drugs	2502 (89.17)	2891 (67.06)	0.000	3974 (89.36)	4763 (70.75)	0.000	7656 (75.85)	6806 (37.98)	0.000	7553 (75.80)	6721 (39.38)	0.000

*Note*: All categorical variables are reported as number (proportion, %) and continuous variables as mean (standard deviation). Cohort of people without missing data on age, sex, ethnicity, IMD, systolic BP, smoking status, BMI (Figures S1 and S2). Missing data for total cholesterol and alcohol intake are reported in Table S1. *p*‐values <0.05 indicate statistical significance.

Abbreviations: BMI, body mass index; BP, blood pressure; COPD, chronic obstructive pulmonary disease; HbA1c, haemoglobin A1c; IMD, index of multiple deprivation; IQR, interquartile range; *n*, number of people.

### 3.2 Mortality in people with ischaemic HF

In the 18 296 individuals with ischaemic HF, during 59 963 person‐years and a median follow‐up of 2.19 [IQR: 0.64–4.87] years, 9584 (52.4%) deaths occurred: 1586 in women with type 2 diabetes (56.5% of all women with type 2 diabetes) and 2265 in men with type 2 diabetes (50.9%), and 2421 (56.2%) and 3312 (49.2%) in those without diabetes (Table 2).

**TABLE 2:** Sex‐stratified mortality rates in people with ischaemic or non‐ischaemic heart failure.

Heart failure, sex, group	Events/People	Crude IR per 100 person‐years (95% CI)	Age‐standardised IR per 100 person‐years (95% CI)	Crude IRR (95% CI)	Age‐adjusted IRR (95% CI)
Ischaemic heart failure					
Women					
Without diabetes	2421/4311	17.85 (17.04–18.67)	15.08 (14.38–15.78)	REF	REF
With type 2 diabetes	1586/2806	18.67 (17.61–19.72)	19.20 (18.12–20.27)	1.05 (0.97–1.12)	1.27 (1.19–1.37)
Men					
Without diabetes	3312/6732	14.42 (13.86–14.98)	16.47 (15.86–17.07)	REF	REF
With type 2 diabetes	2265/4447	15.16 (14.46–15.85)	20.43 (19.48–21.38)	1.05 (0.99–1.11)	1.24 (1.17–1.32)
Non‐ischaemic heart failure					
Women					
Without diabetes	10 485/17921	19.63 (19.19–20.06)	16.59 (16.21–16.98)	REF	REF
With type 2 diabetes	6108/10094	20.03 (19.44–20.61)	19.53 (18.96–20.09)	1.02 (0.98–1.06)	1.18 (1.14–1.22)
Men					
Without diabetes	9566/17069	18.00 (17.58–18.42)	19.35 (18.92–19.77)	REF	REF
With type 2 diabetes	5641/9964	17.74 (17.21–18.26)	21.97 (21.35–22.59)	0.99 (0.95–1.02)	1.14 (1.10–1.18)

*Note*: Age‐standardised rates were estimated at a mean age of the total population of 78 years old.

Abbreviations: CI, confidence interval; IR, incidence rate; IRR, incidence rate ratio; REF, reference group.

**FIGURE 1:**
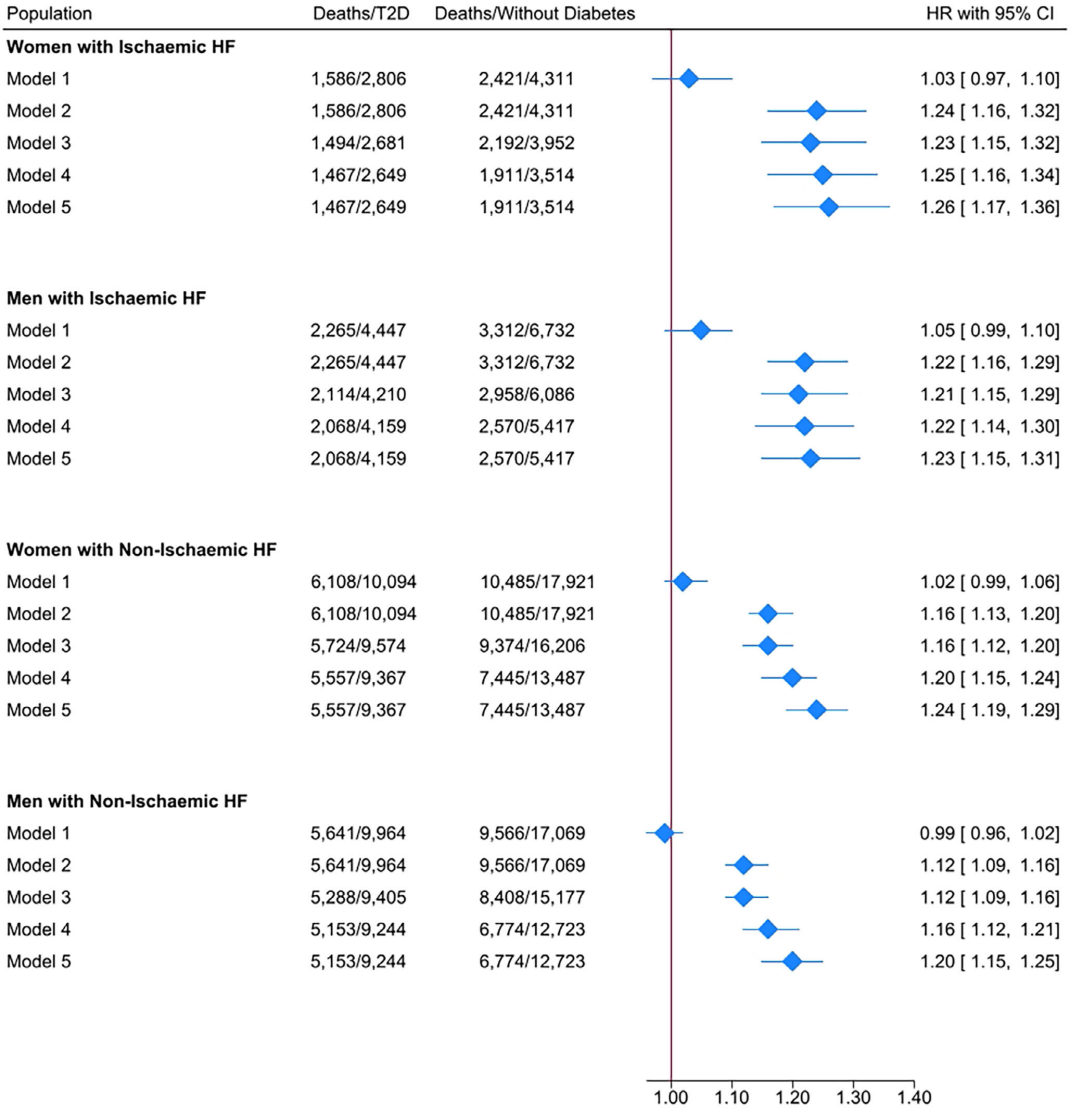
Hazard ratios of mortality Caption/Legend: Reference group for women: Women without diabetes; reference group for men: Men without diabetes. Model (1) Unadjusted. Model (2) Adjusted for age. Model (3) Adjusted for age, index of multiple deprivation, ethnicity, smoking and alcohol intake status. Model (4) Adjusted for age, index of multiple deprivation, ethnicity, smoking, alcohol intake status and comorbidities (anaemia, asthma, atrial fibrillation, cancer, chronic kidney disease, chronic liver disease, chronic obstructive pulmonary disease, dementia, depression, hypertension, osteoarthritis, rheumatoid arthritis, thyroid disorders), body mass index, systolic blood pressure, total cholesterol level. Model (5) Adjusted for age, index of multiple deprivation, ethnicity, smoker status, alcohol intake status, comorbidities (anaemia, asthma, atrial fibrillation, cancer, chronic kidney disease, chronic liver disease, chronic obstructive pulmonary disease, dementia, depression, hypertension, osteoarthritis, rheumatoid arthritis, thyroid disorders), body mass index, systolic blood pressure, total cholesterol level and prescriptions (antihypertensive medications, antiplatelets medications, digoxin, and lipid‐lowering medications). CI, confidence interval; HF, heart failure; HR, hazard ratio; T2D, number of people with type 2 diabetes.

In women, crude rates were similar among people with type 2 diabetes and without diabetes: 18.7 (95% CI: 17.6–19.7) and 17.9 (17.0–18.7) per 100 person‐years, respectively (Table 2); corresponding age‐standardised rates were 19.2 (18.1–20.3) and 15.1 (14.4–15.8), respectively. This translated into crude and age‐adjusted IRRs of death of 1.05 (0.97–1.12) and 1.27 (1.19–1.37) comparing women with type 2 diabetes vs. those without diabetes. For the same comparison, the fully adjusted model (model 5) found a similar result: HR 1.26 (1.17–1.36) (Figure 1).

In men, crude rates were also similar in people with type 2 diabetes and without diabetes: 15.2 (95% CI: 14.5–15.9) and 14.4 (13.9–15.0) per 100 person‐years, respectively, with corresponding age‐standardised rates of 20.4 (19.5–21.4) and 16.5 (15.9–17.1) (Table 2). Comparing men with type 2 diabetes vs. those without diabetes, the crude mortality IRRs were 1.05 (0.99–1.11) while the age‐adjusted IRRs were 1.24 (1.17–1.32). In the fully adjusted model, the HR associated with type 2 diabetes was 1.23 (1.15–1.31) (Figure 1). Model comparison using the likelihood ratio test suggests that there was no interaction between diabetes status and sex in relation to all‐cause mortality risk among people with ischaemic HF (*p* = 0.51).

### 3.3 Mortality in people with non‐ischaemic HF

In the 55 048 people with non‐ischaemic HF, during a median [IQR] follow‐up of 1.98 [0.54–4.53] years and 168 884 person‐years, 31 800 (57.8%) deaths occurred: 6108 in women with type 2 diabetes (60.5% of all women with type 2 diabetes) and 5.641 (56.6%) in men with type 2 diabetes, and 10 485 (58.5%) and 9566 (56.0%), respectively, in those without diabetes (Table 2).

Among women, crude rates were 20.0 (95% CI: 19.4–20.6) and 19.6 (19.2–20.1) per 100 person‐years in those with type 2 diabetes and without diabetes, respectively (Table 2); corresponding age‐standardised rates were 19.5 (19.0–20.1) and 16.6 (16.2–17.0), respectively. Comparing women with type 2 diabetes vs. without diabetes, the crude mortality IRRs were 1.02 (0.98–1.06) while the age‐adjusted IRRs resulted in 1.18 (1.14–1.22). Upon adjustment for several confounders (model 5), the HR associated with type 2 diabetes was 1.24 (1.19–1.29) (Figure 1).

In men, the crude mortality rate was 17.7 (17.2–18.3) per 100 person‐years in people with type 2 and 18.0 (17.6–18.4) in those without diabetes, while corresponding age‐standardised rates were 22.0 (21.4–22.6) and 19.3 (18.9–19.8) (Table 2). These estimates resulted in a crude mortality IRRs of 0.99 (0.95–1.02) and in age‐adjusted IRRs of 1.14 (1.10–1.18) comparing those with type 2 diabetes vs. those without diabetes. In model 5, the HR comparing people with type 2 diabetes to no diabetes was 1.20 (1.15–1.25) (Figure 1). Model comparison using the likelihood ratio test suggests that there was no interaction between diabetes status and sex in relation to all‐cause mortality risk among people with non‐ischaemic HF (*p* = 0.13).

### 3.4 Sensitivity analysis

After including people with prevalent IHD at the diagnosis date of type 2 diabetes (or corresponding date for those without diabetes), our results were consistent with the main analysis, though with attenuated IRRs (Table S4) and HRs (Table S5). Results remained consistent across calendar years of HF diagnosis (Table S6).

## 4 DISCUSSION

This large retrospective cohort study of 73 344 individuals found that people with type 2 diabetes and ischaemic HF had higher mortality rates compared to those without diabetes, in both men and women. Similarly, people with type 2 diabetes and non‐ischaemic HF had higher mortality rates compared to those without diabetes, regardless of sex. Overall, the highest mortality rates were observed in men with type 2 diabetes following a non‐ischaemic HF event. After accounting for several potential confounders, type 2 diabetes was associated with similar excess rates across the two aetiological pathways of HF, in both men and women: for ischaemic HF, ~26% increased rates in women and ~23% in men; for non‐ischaemic HF, ~24% and ~20%, respectively. Furthermore, our results inferred no interaction between diabetes status and sex in relation to all‐cause mortality risk among people with ischaemic and non‐ischaemic HF. Our results therefore suggest that efforts to reduce premature mortality in people with HF should prioritise those with type 2 diabetes, regardless of HF aetiology or sex.

There are limited recent studies that have investigated the risk of death in people with and without diabetes following a diagnosis of ischaemic HF or non‐ischaemic HF. A 2008 study by MacDonald et al. reported that, in 7599 people with HF, diabetes was an independent risk factor for all‐cause mortality, compared to those without diabetes (116 vs. 73 per 1000 person‐years of follow‐up); this study found no interaction between diabetes and sex or ischaemic HF aetiology.^18^ A later 2021 cohort by Tomasik et al. reported that, among those following coronary intervention surgery – a proxy for severe IHD – 75% of the people with diabetes and HF died during the follow‐up period.^24^ In comparison, in our study we found that in individuals with ischaemic HF, 53% and 52% of those with and without type 2 diabetes, respectively, died during the follow‐up; corresponding estimates for non‐ischaemic HF were 59% and 57%. However, Tomasik et al. did not report rates or HR to allow a more direct comparison, used data from a single centre study and excluded those with less severe IHD, which limits representativeness to the general population and comparability with our results. Additionally, a 2016 Swedish study by Johansson et al. reports lower mortality risks compared to our estimates (in people with ischaemic HF, 50% and 43%, in those with and without type 2 diabetes, respectively; and 40% and 34% among those with non‐ischaemic HF, respectively).^17^ Potential reasons for this disparity could be due to differences in the duration of the follow‐up and baseline demographic characteristics, including age and comorbidities.^17^

Our findings are consistent with a 2013 UK study by Cubbon et al., reporting no interaction between diabetes and HF aetiology (ischaemic vs. non‐ischaemic) in their association with all‐cause mortality.^19^ However, the study had a small sample size (*n* = 1091). In addition, the data were from four UK hospitals, which restricts its generalisability to the broader population; in our study, we enhanced the representativeness by using both primary care and hospital data. The Swedish study by Johansson et al. also reported no statistically significant difference in the HR of mortality for ischaemic and non‐ischaemic HF comparing people with type 2 diabetes vs. without diabetes (ischaemic HF: 1.34 [95% CI: 1.24–1.44]; non‐ischaemic HF: 1.21 [1.09–1.34]).^17^ Both these studies utilised data from older cohorts (the UK: 2006–2011; Sweden: 2003–2011), and the results are in line with our findings, which included more up‐to‐date data (2000–2021). In contrast, a 2005 study by Varela et al. found a higher relative risk (RR) among those with non‐ischaemic HF (RR: 1.63) vs. ischaemic HF (RR: 1.18), comparing people with diabetes vs. without diabetes using data from a single centre hospital study between 1991 and 2002.^20^

Although not directly comparable with our study, a recent 2023 study by Yang et al. found that among people with HF with reduced ejection fraction (HFrEF), people with diabetes had a higher HR of mortality (HR: 1.28; 95% CI: 1.07–1.53) compared to those without diabetes.^6^ Similarly, in a recent 2024 cohort study by Gierula et al., diabetes has been identified as a risk factor for mortality in those with HFrEF but not in those with heart failure with preserved ejection fraction (HFpEF).^9^ However, these two studies collected data from single centres with small sample sizes (*n* = 778 and *n* = 3273, respectively); furthermore, the type of diabetes was not reported, whereas our study specifically focused on type 2 diabetes. In contrast, a 2020 cohort study by Al‐Jarallah et al. reported no significant differences in the 3‐month and 12‐month all‐cause mortality comparing people with diabetes vs. without diabetes following an acute HF episode, across different left ventricular ejection fractions. More comprehensive research incorporating both ejection fraction and HF aetiology, rather than considering each factor independently, is needed among people with type 2 diabetes to add greater detail and potentially improve risk stratification.

Our study had several strengths, including the use of CPRD GOLD, CPRD Aurum^21^, ^22^ and HES APC databases to capture population‐level cohorts with incident HF using both primary care and hospital data. The inclusion of nonoverlapping data from both databases enabled us to analyse a large cohort and stratify the population by HF aetiology, diabetes status and sex. Our results contribute to the existing literature, which lacks contemporary data investigating the prognosis following a diagnosis of HF by both HF aetiology and type 2 diabetes status, thus providing more up‐to‐date insights. These findings could therefore be valuable for informing future policy decisions and designing targeted interventions aimed at reducing premature mortality in individuals with HF and type 2 diabetes, such as more frequent clinical assessments or type 2 diabetes‐specific targets for the treatment of HF. However, our study has several limitations. Firstly, we relied on routinely collected health records, which may have introduced measurement errors for some covariates. Secondly, due to the large sample size, imputing missing data was computationally demanding, so we opted for a complete case analysis. Thirdly, the absence of echocardiography data meant we could not distinguish between HF with preserved or reduced ejection fraction. Fourthly, the increasing trend in NT‐proBNP testing in the United Kingdom between 2004 and 2018 might have altered the number of HF cases identified in our study.^25^ Moreover, the current findings should be interpreted in view of the increasing use of CT coronary angiography in the United Kingdom between 2011 and 2017.^26^ This might have contributed to higher detection rates of IHD over time and, consequently, the number of people with ischaemic HF identified in our study. Additionally, we acknowledge that changes in prescription trends for diabetes and cardiovascular disease medications in the United Kingdom could have influenced our results.^27^ Furthermore, the authors acknowledge that the presence of other cardiovascular conditions could have contributed to misclassification bias of incident ischaemic and non‐ischaemic HF. For example, individuals diagnosed with prevalent arrhythmia, valvular heart disease or cardiomyopathies who later develop incident IHD and HF may have been misclassified as having ischaemic HF, potentially overestimating the number of people diagnosed with ischaemic HF in the study. Lastly, certain confounding variables, such as body fat distribution, physical activity and dietary factors, were either not available or not well recorded in the CPRD and HES databases, for which residual confounding cannot be ruled out in a more aetiologically focused interpretation of our results.

In conclusion, our study showed higher mortality rates following a diagnosis of HF in those with type 2 diabetes vs. without diabetes, regardless of sex or HF aetiology, with the excess mortality associated with type 2 diabetes similar following ischaemic or non‐ischaemic HF. This highlights the ongoing need to optimise the prevention and management strategies in the overall population of individuals with type 2 diabetes and HF to reduce the risk of premature mortality.

## AUTHOR CONTRIBUTIONS

KP contributed to the conception and design of work, data cleaning, data analysis, validation and interpretation of results, including drafting the original article and revising the draft for important intellectual content. CL and FZ contributed to the conception and design of work, supervision, data cleaning, data analysis, validation and interpretation of the results, including revising the draft for important intellectual content. SS contributed to the data cleaning and analysis, interpretation of the results and revising the draft for important intellectual content. KK contributed to the conception and design of work, interpretation of the results and revising the draft for important intellectual content.

## FUNDING INFORMATION

NIHR Applied Research Collaboration East Midlands has funded this study. The views expressed are those of the author(s) and not necessarily those of the NIHR or the Department of Health and Social Care. The funders had no role in the conception of the study design or in the collection process, data analysis, interpretation of data, writing of the report or decision to submit the article for publication in Diabetes, Obesity and Metabolism.

## CONFLICT OF INTEREST STATEMENT

All authors have completed the ICMJE uniform disclosure form at www.icmje.org/coi_disclosure.pdf. KP declares this research was supported by the NIHR ARC EM. FZ, CL and SS are supported from the NIHR ARC EM and NIHR Leicester Biomedical Research Centre. FZ: consultancy for Servier, Daiichi Sankyo, Menarini. KK has acted as a consultant, speaker or received grants for investigator‐initiated studies for Astra Zeneca, Bayer, Novo Nordisk, Sanofi‐Aventis, Servier, Lilly and Merck Sharp & Dohme, Boehringer Ingelheim, Oramed Pharmaceuticals, Pfizer, Roche, Daiichi Sankyo, Applied Therapeutics, Embecta and Nestle Health Science.

## PEER REVIEW

The peer review history for this article is available at https://www.webofscience.com/api/gateway/wos/peer‐review/10.1111/dom.16413.

## Supporting information


**Data S1.** Supporting Information.

## Data Availability

Data is available by approval from CPRD only.
