# Supplementary material for: Mortality risk following ischaemic and non‐ischaemic heart failure in people with type 2 diabetes: Observational study in England, 2000–2021
Source: Diabetes Obes Metab. 2025 May 2;27(7):3848–57. doi: 10.1111/dom.16413 (PMC12146457; doi:10.1111/dom.16413)
Supplement: Supplementary file 1 — Data S1. Supporting Information. [file DOM-27-3848-s001.docx]

**SUPPLEMENTARY MATERIAL**

**Mortality risk following ischaemic and non-ischaemic heart failure in people with type 2 diabetes:**

**observational study in England, 2000-2021**

**Contents**

[**Table S1: Missing data at baseline (heart failure diagnosis)** 2](#_Toc194317850)

[**Table S2: Number of individuals with heart failure diagnosis by sex, type 2 diabetes, and year of diagnosis** 3](#_Toc194317851)

[**Table S3: Duration from type 2 diabetes diagnosis (or corresponding date for those without diabetes) to incident ischaemic heart disease, and from ischaemic heart disease to incident heart failure.** 4](#_Toc194317852)

[**Table S4: Sensitivity analysis – sex-stratified mortality rates in individuals with ischaemic heart failure** 5](#_Toc194317853)

[**Table S5: Sensitivity analysis – sex-stratified hazard ratios for mortality in individuals with ischaemic heart failure** 6](#_Toc194317854)

[**Table S6: Sensitivity analysis – sex-stratified mortality rates in individuals with ischaemic and non-ischaemic heart failure at study entry in 2000-2010 or 2011-2021** 7](#_Toc194317855)

[**Figure S1: Flowchart of cohort definition in CPRD GOLD** 8](#_Toc194317856)

[**Figure S2: Flowchart of cohort definition in CPRD Aurum** 9](#_Toc194317857)

[**RECORD CHECKLIST** 10](#_Toc194317858)

#

# **Table S1: Missing data at baseline (heart failure diagnosis)**

|  | **Ischaemic heart failure** | | | | | | **Non-ischaemic heart failure** | | | | | |
| --- | --- | --- | --- | --- | --- | --- | --- | --- | --- | --- | --- | --- |
|  | **Total population**  **n = 18,296** | | **Type 2 diabetes**  **n =** **7,253** | | **Without diabetes  n = 11,043** | | **Total population**  **n = 55,048** | | **Type 2 diabetes**  **n = 20,058** | | **Without diabetes  n = 34,990** | |
|  | **Missing (n)** | **Missing (%)** | **Missing (n)** | **Missing (%)** | **Missing (n)** | **Missing (%)** | **Missing (n)** | **Missing (%)** | **Missing (n)** | **Missing (%)** | **Missing (n)** | **Missing (%)** |
| Total cholesterol | 1,463 | 8.00 | 109 | 1.50 | 1,354 | 12.26 | 6,791 | 12.34 | 451 | 2.25 | 6,340 | 18.12 |
| Alcohol intake status | 1,367 | 7.47 | 362 | 4.99 | 1,005 | 9.10 | 4,686 | 8.51 | 1,079 | 5.38 | 3,607 | 10.31 |

Abbreviations: n = number of people.

Missing data for total cholesterol and alcohol intake status in the cohort of people without missing data on ethnicity, IMD, systolic BP, smoking status, BMI (Figure S1 and S2).

# **Table S2: Number of individuals with heart failure diagnosis by sex, type 2 diabetes, and year of diagnosis**

|  | **Ischaemic heart failure** | | | | **Non-ischaemic heart failure** | | | |
| --- | --- | --- | --- | --- | --- | --- | --- | --- |
| **Year of heart failure diagnosis** | **Women with type 2 diabetes  n = 2,806** | **Women without diabetes   n = 4,311** | **Men with type 2 diabetes  n = 4,447** | **Men without diabetes  n = 6,732** | **Women with type 2 diabetes n = 10,094** | **Women without diabetes  n = 17,921** | **Men with type 2 diabetes  n = 9,964** | **Men without diabetes  n = 17,069** |
| 2000 | * | * | * | * | * | 21 | * | 25 |
| 2001 | * | * | * | * | 64 | 66 | 51 | 84 |
| 2002 | * | 26 | 28 | 25 | 100 | 140 | 68 | 116 |
| 2003 | 35 | 42 | 41 | 65 | 117 | 216 | 142 | 191 |
| 2004 | 34 | 44 | 73 | 70 | 158 | 254 | 147 | 225 |
| 2005 | 59 | 61 | 64 | 100 | 207 | 306 | 194 | 294 |
| 2006 | 48 | 80 | 92 | 115 | 219 | 312 | 231 | 363 |
| 2007 | 62 | 72 | 87 | 148 | 264 | 436 | 237 | 443 |
| 2008 | 77 | 132 | 111 | 175 | 283 | 497 | 292 | 469 |
| 2009 | 90 | 155 | 132 | 231 | 369 | 565 | 352 | 548 |
| 2010 | 122 | 152 | 182 | 273 | 409 | 690 | 383 | 625 |
| 2011 | 138 | 224 | 162 | 298 | 442 | 744 | 446 | 767 |
| 2012 | 163 | 224 | 211 | 384 | 514 | 929 | 485 | 838 |
| 2013 | 149 | 268 | 258 | 395 | 584 | 1,059 | 523 | 1,047 |
| 2014 | 155 | 284 | 286 | 432 | 633 | 1,191 | 618 | 1,187 |
| 2015 | 200 | 340 | 325 | 506 | 751 | 1,413 | 759 | 1,223 |
| 2016 | 238 | 334 | 379 | 541 | 821 | 1,550 | 785 | 1,341 |
| 2017 | 252 | 437 | 430 | 630 | 886 | 1,550 | 934 | 1,538 |
| 2018 | 245 | 440 | 470 | 676 | 997 | 1,758 | 987 | 1,672 |
| 2019 | 346 | 470 | 529 | 791 | 1,070 | 1,970 | 1,108 | 1,946 |
| 2020 | 297 | 414 | 458 | 693 | 941 | 1,782 | 975 | 1,703 |
| 2021 | 68 | 101 | 111 | 168 | 248 | 472 | 230 | 424 |

* In columns with cells with frequency ≤5, cell suppression has been applied if frequency ≤20.

# **Table S3: Duration from type 2 diabetes diagnosis (or corresponding date for those without diabetes) to incident ischaemic heart disease, and from ischaemic heart disease to incident heart failure.**

| **Sex, group** | **Median (IQR) duration (years) between diagnosis of type 2 diabetes (corresponding date for those without diabetes) to incident ischaemic heart disease** | **Median (IQR) duration (years) between diagnosis of ischaemic heart disease to incident heart failure** |
| --- | --- | --- |
| *Total*  People without diabetes  People with type 2 diabetes | 5.07 (2.12-9.03)  5.27 (2.32-9.08)  4.70 (1.83-8.92) | 1.17 (0.10-5.37) 1.14 (0.10-5.38)  1.21 (0.10-5.34) |
| *Women*  People without diabetes  People with type 2 diabetes | 5.20 (2.16-9.09)  5.41 (2.42-9.30)  4.66 (1.84-8.76) | 1.28 (0.10-5.57)  1.26 (0.09-5.41)  1.33 (0.12-5.86) |
| *Men*  People without diabetes  People with type 2 diabetes | 5.00 (2.10-8.99)  5.16 (2.28-8.97)  4.73 (1.82-9.04) | 1.08 (0.10-5.24)  1.05 (0.10-5.37)  1.13 (0.10-5.06) |

Abbreviations: IQR = interquartile range.

# **Table S4: Sensitivity analysis – sex-stratified mortality rates in individuals with ischaemic heart failure**

| **Sex, group** | **Events/N** | **Crude IR per 100 person-years (95% CI)** | **Age-standardised IR per 100 person-years (95% CI)** | **Crude IRR  (95% CI)** | **Age-adjusted IRR**  **(95% CI)** |
| --- | --- | --- | --- | --- | --- |
| *Women*   Without diabetes  With type 2 diabetes | 6,083/9,724 4,667/7,445 | 20.06 (19.48–20.64)  20.22 (19.55–20.89) | 16.14 (15.66–16.62) 18.90 (18.28–19.52) | REF  1.01 (0.96–1.05) | REF  1.17 (1.12–1.22) |
| *Men*  Without diabetes  With type 2 diabetes | 9,166/15,912  7,379/12,606 | 17.24 (16.84–17.65)  17.10 (16.66–17.54) | 18.24 (17.83–18.64) 20.72 (20.21–21.23) | REF 0.99 (0.96–1.03) | REF 1.14 (1.10–1.17) |

Compared to the main analysis (Table 2), this sensitivity analysis still excluded individuals with prevalent peripheral vascular disease or stroke but included those with prevalent ischaemic heart disease prior to or at the diagnosis date of type 2 diabetes (or the corresponding date in those without diabetes).

Abbreviations: N = number of people, IR = incidence rate, IRR = incidence rate ratio, CI = confidence interval, REF = reference group.

Age–standardised rates were estimated at mean age of the total population at 78 years old.

# **Table S5: Sensitivity analysis – sex-stratified hazard ratios for mortality in individuals with ischaemic heart failure**

| **Sex** | **Model 1** | | **Model 2** | | **Model 3** | | **Model 4** | | **Model 5** | |
| --- | --- | --- | --- | --- | --- | --- | --- | --- | --- | --- |
|  | **Events/N** | **HR (95% CI)** | **Events/N** | **HR (95% CI)** | **Events/N** | **HR (95% CI)** | **Events/N** | **HR (95% CI)** | **Events/N** | **HR (95% CI)** |
| Women Men | 10,750/17,169  16,545/28,518 | 1.01 (0.97–1.04) 1.00 (0.97–1.03) | 10,750/17,169 16,545/28,518 | 1.15 (1.11–1.19) 1.13 (1.09–1.16) | 9,967/16,060  15,172/26,384 | 1.14 (1.09–1.18) 1.13 (1.09–1.17) | 9,285/15,135  14,314/25,071 | 1.17 (1.12–1.22) 1.15 (1.11–1.19) | 9,285/15,135  14,314/25,071 | 1.17 (1.12–1.23) 1.16 (1.12–1.20) |

Compared to the main analysis (Table 3), this sensitivity analysis still excluded individuals with prevalent peripheral vascular disease or stroke but included those with prevalent ischaemic heart disease prior to or at the diagnosis date of type 2 diabetes (or the corresponding date in those without diabetes).

Model 1) Unadjusted.

Model 2) Adjusted for age.

Model 3) Adjusted for age, index of multiple deprivation, ethnicity, smoking, and alcohol intake status.
Model 4) Adjusted for age, index of multiple deprivation, ethnicity, smoking, alcohol intake status and comorbidities (anaemia, asthma, atrial fibrillation, cancer, chronic kidney disease, chronic liver disease, chronic obstructive pulmonary disease, dementia, depression, hypertension, osteoarthritis, rheumatoid arthritis, thyroid disorders), body mass index, systolic blood pressure, total cholesterol level.
Model 5) Adjusted for age, index of multiple deprivation, ethnicity, smoker status, alcohol intake status, comorbidities (anaemia, asthma, atrial fibrillation, cancer, chronic kidney disease, chronic liver disease, chronic obstructive pulmonary disease, dementia, depression, hypertension, osteoarthritis, rheumatoid arthritis, thyroid disorders), body mass index, systolic blood pressure, total cholesterol level and prescriptions (antihypertensive medications, antiplatelets medications, digoxin, and lipid–lowering medications).

Abbreviations: HR = hazard ratio, CI = confidence intervals, N = number of people.

# **Table S6: Sensitivity analysis – sex-stratified mortality rates in individuals with ischaemic and non-ischaemic heart failure at study entry in 2000-2010 or 2011-2021**

| **Sex, group** | **Age-standardised IR**  **per 100 person-years (95% CI)** | **Age-adjusted IRR**  **(95% CI)** | **Model 5**  **HR (95%CI)** |
| --- | --- | --- | --- |
| **Diagnosis of incident heart failure in years 2000-2010** | | | |
| **Ischaemic HF** | | | |
| *Women*   Without diabetes  With type 2 diabetes | 15.16 (13.97-16.35)   19.46 (17.56-21.36) | REF  1.28 (1.13-1.46) | REF 1.24 (1.07-1.43) |
| *Men*  Without diabetes  With type 2 diabetes | 15.83 (14.74-16.91)  19.38 (17.62-21.15) | REF 1.22 (1.10-1.37) | REF  1.17 (1.03-1.32) |
| **Non-ischaemic HF** | | | |
| *Women*   Without diabetes  With type 2 diabetes | 16.29 (15.65-16.93)  18.99 (18.08-19.89) | REF 1.17 (1.10-1.24) | REF 1.28 (1.19-1.38) |
| *Men*  Without diabetes  With type 2 diabetes | 19.77 (18.99-20.54)  22.37 (21.25-23.49) | REF 1.13 (1.06-1.21) | REF  1.20 (1.11-1.29) |
| **Diagnosis of incident heart failure in years 2011-2021** | | | |
| **Ischaemic HF** |  |  |  |
| *Women*   Without diabetes  With type 2 diabetes | 15.01 (14.13-15.89)  19.04 (17.72-20.36) | REF 1.27 (1.17-1.38) | REF 1.24 (1.14-1.35) |
| *Men*  Without diabetes  With type 2 diabetes | 16.63 (15.90-17.36)  20.77 (19.64-21.89) | REF 1.25 (1.16-1.34) | REF 1.25 (1.16-1.34) |
| **Non-ischaemic HF** |  |  |  |
| *Women*   Without diabetes  With type 2 diabetes | 16.71 (16.22-17.19)  19.77 (19.05-20.48) | REF 1.18 (1.13-1.24) | REF 1.21 (1.16-1.26) |
| *Men*  Without diabetes  With type 2 diabetes | 19.05 (18.54-19.57)  21.66 (20.90-22.41) | REF 1.14 (1.09-1.19) | REF 1.18 (1.12-1.23) |

Abbreviations: HR = hazard ratio, IR = incidence rate, IRR = incidence rate ratio, CI = confidence interval, REF = reference group.

Age–standardised rates were estimated at mean age of the total population at 78 years old.

Model 5) Adjusted for age, index of multiple deprivation, ethnicity, smoker status, alcohol intake status, comorbidities (anaemia, asthma, atrial fibrillation, cancer, chronic kidney disease, chronic liver disease, chronic obstructive pulmonary disease, dementia, depression, hypertension, osteoarthritis, rheumatoid arthritis, thyroid disorders), body mass index, systolic blood pressure, total cholesterol level and prescriptions (antihypertensive medications, antiplatelets medications, digoxin, and lipid–lowering medications).

# **Figure S1: Flowchart of cohort definition in CPRD GOLD**


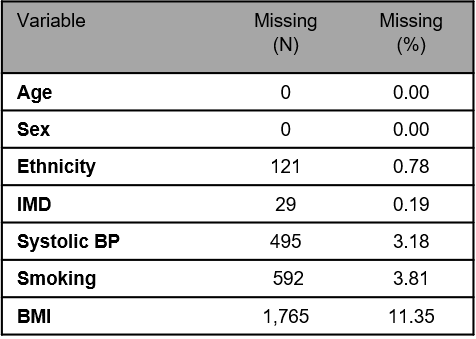


N = 33,950
(T2D = 13,471; without diabetes = 20,479**)**

N = 16,092
(T2D = 5,289; without diabetes = 10,803)

N = 15,544
(T2D = 5,101; without diabetes = 10,443)

Excluded those prescribed glucose-lowering drugs and without diabetes = 21

Excluded if date of death or end date is at or prior to 1^st^ HF diagnosis date = 548

Excluded HF or CVD (MI, IHD, stroke, angina, PVD) at or prior to type 2 diabetes diagnosis date (corresponding date for people without diabetes) = 17,837

N = 16,113
(T2D = 5,289; without diabetes = 10,824)

**CPRD GOLD participants aged ≥18 years old between 1^st^ January 2000 until 29^th^ March 2021
N = 1,057,998 (T2D = 214,640; without diabetes = 843,358)**

Excluded those without an incident HF diagnosis during follow-up = 1,024,048

**N = 13,536
(T2D = 4,823; without diabetes = 8,713)**

Abbreviations: BMI = body mass index, BP = blood pressure, CPRD = Clinical Research Practice datalink data, CVD = cardiovascular disease, HF = heart failure, IHD = ischaemic heart disease, IMD = index of multiple deprivation, MI = myocardial infarction, N = total participants, PVD = peripheral vascular disease, T2D = type 2 diabetes. End date is 29^th^ March 2021.

# **Figure S2: Flowchart of cohort definition in CPRD Aurum**


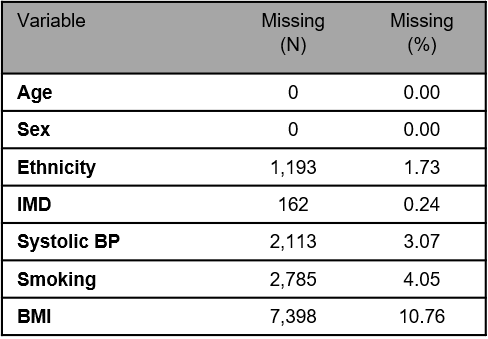


**N = 59,808
(T2D = 22,488; without diabetes = 37,320)**

N = 136,040
(T2D = 55,976; without diabetes = 80,064)

N = 71,277
(T2D = 24,740; without diabetes = 46,537)

N = 68,774
(T2D = 23,825; without diabetes = 44,949)

Excluded those prescribed glucose-lowering drugs and without diabetes = 249

Excluded if date of death or end date is at or prior to 1^st^ HF diagnosis date = 2,503

Excluded HF or CVD (MI, IHD, stroke, angina, PVD) at or prior to type 2 diabetes diagnosis date (corresponding date for people without diabetes) = 64,514

N = 71,526
(T2D = 24,740; without diabetes = 46,786)

**CPRD Aurum participants aged ≥18 years old between 1^st^ January 2000 until 29^th^ March 2021
N = 3,538,922 (T2D = 722,432; without diabetes = 2,816,490)**

Excluded those without an incident HF diagnosis during follow-up = 3,402,882

Abbreviations: BMI = body mass index, BP = blood pressure, CPRD = Clinical Research Practice datalink data, CVD = cardiovascular disease, HF = heart failure, IHD = ischaemic heart disease, IMD = index of multiple deprivation, MI = myocardial infarction, N = total participants, PVD = peripheral vascular disease, T2D = type 2 diabetes. End date is 29^th^ March 2021.

# **RECORD CHECKLIST**

|  | **Item No.** | **STROBE items** | **Location in manuscript where items are reported** | **RECORD items** | **Location in manuscript where items are reported** |
| --- | --- | --- | --- | --- | --- |
| **Title and abstract** | | | | | |
|  | 1 | (a) Indicate the study’s design with a commonly used term in the title or the abstract (b) Provide in the abstract an informative and balanced summary of what was done and what was found | Page 1–2 | RECORD 1.1: The type of data used should be specified in the title or abstract. When possible, the name of the databases used should be included.  RECORD 1.2: If applicable, the geographic region and timeframe within which the study took place should be reported in the title or abstract.  RECORD 1.3: If linkage between databases was conducted for the study, this should be clearly stated in the title or abstract. | Page 1–2  Page 1–2  Page 1–2 |
| **Introduction** | | | | | |
| Background rationale | 2 | Explain the scientific background and rationale for the investigation being reported | Page 3 |  |  |
| Objectives | 3 | State specific objectives, including any prespecified hypotheses | Page 3 |  |  |
| **Methods** | | | | | |
| Study Design | 4 | Present key elements of study design early in the paper | P Page 4 |  |  |
| Setting | 5 | Describe the setting, locations, and relevant dates, including periods of recruitment, exposure, follow–up, and data collection | Page 4–5 |  |  |
| Participants | 6 | *(a) Cohort study* – Give the eligibility criteria, and the sources and methods of selection of participants. Describe methods of follow–up  *(b) Cohort study* – For matched studies, give matching criteria and number of exposed and unexposed | Page 4–5  Page 4; Figure S1; Figure S2 | RECORD 6.1: The methods of study population selection (such as codes or algorithms used to identify subjects) should be listed in detail. If this is not possible, an explanation should be provided.  RECORD 6.2: Any validation studies of the codes or algorithms used to select the population should be referenced. If validation was conducted for this study and not published elsewhere, detailed methods and results should be provided.  RECORD 6.3: If the study involved linkage of databases, consider use of a flow diagram or other graphical display to demonstrate the data linkage process, including the number of individuals with linked data at each stage. | Page 4–5;  Figure S1; Figure S2  Page 4  Page 4–5 |
| Variables | 7 | Clearly define all outcomes, exposures, predictors, potential confounders, and effect modifiers. Give diagnostic criteria, if applicable. | Page 4–5 | RECORD 7.1: A complete list of codes and algorithms used to classify exposures, outcomes, confounders, and effect modifiers should be provided. If these cannot be reported, an explanation should be provided. | Page 4 |
| Data sources/ measurement | 8 | For each variable of interest, give sources of data and details of methods of assessment (measurement).  Describe comparability of assessment methods if there is more than one group | Page 4–5 |  |  |
| Bias | 9 | Describe any efforts to address potential sources of bias | Page 4–5 |  |  |
| Study size | 10 | Explain how the study size was arrived at | Page 4–5;  Figure S1; Figure S2 |  |  |
| Quantitative variables | 11 | Explain how quantitative variables were handled in the analyses. If applicable, describe which groupings were chosen, and why | Page 4–5 |  |  |
| Statistical methods | 12 | (a) Describe all statistical methods, including those used to control for confounding  (b) Describe any methods used to examine subgroups and interactions  (c) Explain how missing data were addressed  (d) *Cohort study* – If applicable, explain how loss to follow–up was addressed  (e) Describe any sensitivity analyses | Page 4–5  Page 5  Page 4-5   Page 4-5  Page 6 |  |  |
| Data access and cleaning methods |  | .. |  | RECORD 12.1: Authors should describe the extent to which the investigators had access to the database population used to create the study population.  RECORD 12.2: Authors should provide information on the data cleaning methods used in the study. | Page 4-5;  Page 12-11  Page 4-5 |
| Linkage |  | .. |  | RECORD 12.3: State whether the study included person–level, institutional–level, or other data linkage across two or more databases. The methods of linkage and methods of linkage quality evaluation should be provided. | Page 4 |
| **Results** | | | | | |
| Participants | 13 | (a) Report the numbers of individuals at each stage of the study (*e.g.*, numbers potentially eligible, examined for eligibility, confirmed eligible, included in the study, completing follow–up, and analysed)  (b) Give reasons for non–participation at each stage.  (c) Consider use of a flow diagram | Figure S1 and Figure S2 | RECORD 13.1: Describe in detail the selection of the persons included in the study (*i.e.,* study population selection) including filtering based on data quality, data availability and linkage. The selection of included persons can be described in the text and/or by means of the study flow diagram. | Page 4-7; Figure S1;  Figure S2 |
| Descriptive data | 14 | (a) Give characteristics of study participants (*e.g.*, demographic, clinical, social) and information on exposures and potential confounders  (b) Indicate the number of participants with missing data for each variable of interest  (c) *Cohort study* – summarise follow–up time (*e.g.*, average and total amount) | Page 7-8;  Table 1; Table S1  Figure 1    Table S1    Page 7-8; |  |  |
| Outcome data | 15 | *Cohort study* – Report numbers of outcome events or summary measures over time | Page 7-8;  Table 2; Figure 1 |  |  |
| Main results | 16 | (a) Give unadjusted estimates and, if applicable, confounder–adjusted estimates and their precision (e.g., 95% confidence interval). Make clear which confounders were adjusted for and why they were included.  (b) Report category boundaries when continuous variables were categorized  (c) If relevant, consider translating estimates of relative risk into absolute risk for a meaningful time period | Page 7-8; Table 2;  Figure 1 |  |  |
| Other analyses | 17 | Report other analyses done—e.g., analyses of subgroups and interactions, and sensitivity analyses | Page 8 |  |  |
| **Discussion** | | | | | |
| Key results | 18 | Summarise key results with reference to study objectives | Page 9; Page 11 |  |  |
| Limitations | 19 | Discuss limitations of the study, taking into account sources of potential bias or imprecision. Discuss both direction and magnitude of any potential bias | Page 10-11 | RECORD 19.1: Discuss the implications of using data that were not created or collected to answer the specific research question(s). Include discussion of misclassification bias, unmeasured confounding, missing data, and changing eligibility over time, as they pertain to the study being reported. | Page 9-11 |
| Interpretation | 20 | Give a cautious overall interpretation of results considering objectives, limitations, multiplicity of analyses, results from similar studies, and other relevant evidence | Page 9-11 |  |  |
| Generalisability | 21 | Discuss the generalisability (external validity) of the study results | Page 9-11 |  |  |
| **Other Information** | | | | | |
| Funding | 22 | Give the source of funding and the role of the funders for the present study and, if applicable, for the original study on which the present article is based | Page 12 |  |  |
| Accessibility of protocol, raw data, and programming code |  | .. | Page 4 | RECORD 22.1: Authors should provide information on how to access any supplemental information such as the study protocol, raw data, or programming code. | Page 4 |

Benchimol EI, Smeeth L, Guttmann A, Harron K, Moher D, Petersen I, Sørensen HT, von Elm E, Langan SM, the RECORD Working Committee. The Reporting of studies Conducted using Observational Routinely collected health Data (RECORD) Statement. <https://doi.org/10.1371/journal.pmed.1001885>.

Page numbers refer to the original submission.
